# Normative modernization, social integration, and collective wellbeing: Evidence from a cross-national dynamic panel study

**DOI:** 10.1371/journal.pone.0360799

**Published:** 2026-10-23

**Authors:** Ariz Naqvi

**Affiliations:** College of Business Administration, American University of the Middle East, Egaila, Kuwait; Hunan University, CHINA

## Abstract

Cross-national evidence does not establish whether changing normative environments are associated with population wellbeing independently of social integration. Using repeated International Social Survey Programme data, the reported analysis aggregates individual responses into 286 country-wave observations across 52 countries. Two-step System GMM models estimate persistence and conditional associations while treating the lagged outcome as endogenous. Reported estimates indicate persistence in countries’ relative wellbeing positions. Social integration has a modest positive association with wellbeing, while the composite modernization association is small and imprecise. Gender egalitarianism and environmental concern show modest positive domain-specific associations; inequality attitudes, religiosity, and work modernization do not. The modernization association is more positive at higher integration levels. These findings are conditional associations, not causal effects. Interpretation is restricted by low ICC(1) values, incomplete cross-national measurement invariance, a changing-domain composite, and only three directly observed social-integration waves. The evidence therefore supports a bounded conclusion: aggregate normative indicators are associated with relative wellbeing positions differently across domains and integration levels.

## 1. Introduction

Despite widespread normative change across societies, cross-national evidence linking normative environments to population wellbeing remains inconsistent [1,2]. As collective meaning systems, normative environments provide common expectations about appropriate beliefs, behaviors, and social arrangements, enabling individuals to anticipate others’ actions and coordinate their own behavior [1,3]. When norms are widely shared and socially embedded, they support psychologically relevant conditions such as perceived predictability, security, and fairness features closely linked to wellbeing [4,5]. Conversely, normative environments characterized by ambiguity, fragmentation, or weak embedding can undermine expectation stability and generate uncertainty that erodes psychological stability [6,7]. Clarifying how shared normative climates shape wellbeing represents a central concern across social psychology and applied research on societal wellbeing and institutional functioning [6,8].

Over recent decades, many societies have shifted toward greater egalitarianism, individual autonomy, secularization, and support for institutional fairness [9,10]. These shifts are often described as normative modernization, changes in shared beliefs about social roles, equality, and legitimacy [10,11]. From a social-psychological perspective, normative modernization matters because it alters the collective meaning structures through which individuals evaluate social relations and institutional treatment [2,6]. Normative climates emphasizing fairness and autonomy can strengthen wellbeing by shaping how predictable and legitimate social environments feel, reducing perceived arbitrariness, and increasing perceived alignment between expectations and lived environments [4,12]. Yet cross-national evidence linking normative modernization to wellbeing is mixed, with associations that are often weak, ambiguous, or context-dependent [13,14]. This heterogeneity suggests that normative climates may not carry uniform psychological consequences across societies, but rather operate within broader environments that shape whether shared expectations become stable, credible, and consequential for everyday experience [15,16].

Social integration represents a plausible boundary condition for these effects. Integration captures the extent to which individuals are embedded in cohesive networks characterized by trust, support, and stable interpersonal expectations [17,18]. Integrated societies provide predictable social environments that reduce uncertainty and support coordinated interaction, thereby contributing to psychological stability and collective wellbeing [4,12]. Comparative evidence also links cohesion and integration to higher national wellbeing net of economic conditions [13,19]. Critically, from a climate perspective, integration is not only an individual resource; it is a shared structural environment that affects the credibility, enforceability, and perceived consistency of norms [20,21]. Where integration is high, shared norms are more likely to be perceived as broadly endorsed and reliably upheld, stabilizing expectations and amplifying psychological consequences. Where integration is low, normative change may be less consistently embedded, weakening normative influence and potentially sustaining uncertainty [2,8].

Beyond theoretical debates about normative change, understanding when evolving norms translate into improved societal wellbeing has important applied implications. Societies undergoing rapid value change often confront tensions between emerging expectations and existing coordination environments. Identifying conditions under which normative modernization supports psychological security and collective wellbeing can inform policy discussions concerning social cohesion, institutional legitimacy, and the management of social change.

The analysis treats normative modernization, social integration, and collective wellbeing as country-wave aggregate indicators and estimates their dynamic associations. The term ‘climate’ identifies the theoretical interpretation being evaluated, not an established contextual effect. Repeated cross-sectional ISSP data are aggregated into an unbalanced panel to estimate persistence, direct associations, and the reported interaction while retaining the distinction between aggregate patterns and individual-level mechanisms.

## 2. Literature review

### 2.1. Normative modernization and wellbeing: theoretical promise and empirical ambiguity

Normative modernization occupies a central place in accounts of social change and human wellbeing [10,22]. Classical modernization theory links structural transformation, economic development, educational expansion, and institutional differentiation to value shifts toward autonomy, egalitarianism, secularization, and self-expression [9,23]. These shifts are theorized to support wellbeing by loosening coercive constraints, expanding self-determination, and enabling arrangements that better reflect individual preferences [24]. In this view, normative modernization is a key route through which structural development translates into improved quality of life [14,25].

Human development and cultural change perspectives extend this claim by emphasizing institutional consequences of normative transformation. Normative climates prioritizing equality, autonomy, and fairness are associated with more inclusive arrangements and more legitimate governance environments [8,11,26]. Such environments are expected to support wellbeing by strengthening perceived fairness, institutional trust, and psychological security [5,27]. Accordingly, cross-national research often reports positive associations between egalitarian normative climates and population wellbeing [4,28].

However, evidence is more heterogeneous than linear modernization narratives imply [14,29]. Some societies have experienced normative modernization without sustained gains in collective wellbeing, while others remain relatively traditional yet show high wellbeing [29–31]. This inconsistency has been framed as part of a broader modernization-wellbeing paradox [5,25]. Recent cross-national work likewise suggests that normative modernization does not exert uniform psychological effects across contexts [6,13]. Instead, its consequences appear contingent on whether evolving expectations become embedded in the environments that structure everyday coordination and legitimacy [16,30,32].

A key explanation lies in the distinction between normative orientation and institutional realization [1,3,15]. Normative modernization reflects shared expectations about how social relations should be organized, but such expectations need not translate into lived environments or institutional arrangements [11,33,34]. Normative climates are theorized to influence wellbeing through environments that affect legitimacy, predictability, and stability [7,35]. When expectations align with institutional structures, norms can reinforce coherence and support wellbeing [12,36,37]. When alignment is weaker, modernization may coexist with misaligned environments that blunt its association with wellbeing [6,16].

Empirically, this climate-level interpretation is often not tested. Many studies operationalize modernization as individual-level attitudes and regress them directly on individual wellbeing, treating normative change as a personal attribute rather than as a shared environment that structures coordination and legitimacy [3,38]. This approach is not well suited to evaluating whether shared normative climates shape collective wellbeing via contextual mechanisms. Moreover, modernization is multidimensional; domains such as gender egalitarianism, environmental concern, religiosity, inequality attitudes, and work-related norms may have distinct and potentially offsetting associations with wellbeing, making uniform effects unlikely.

A further limitation concerns temporal dynamics. National wellbeing climates show substantial stability, plausibly reflecting enduring institutional arrangements, cultural continuity, and structural inertia [16,33,39]. Static cross-sectional models risk conflating contemporaneous associations with inherited trajectories because prior institutional environments shape both current wellbeing and current normative climates [40,41]. Dynamic perspectives therefore emphasize evaluating normative modernization within persistent environments and distinguishing continuity from contemporaneous associations [8,42,43].

In sum, the literature provides strong reasons to expect normative modernization to matter for collective wellbeing, but also indicates heterogeneity and modest average effects. This motivates a contextual framework that treats modernization as a multidimensional normative climate, embeds it in structural environments, and models persistence explicitly.

### 2.2. Social integration as a structural determinant of wellbeing climates

Social integration represents a foundational condition shaping collective psychological environments. Classical sociological and psychological perspectives emphasize that individuals derive psychological stability and security from embeddedness in cohesive social environments characterized by stable relationships, shared expectations, and coordinated social interaction [17,44]. Integrated social environments provide predictable interactional contexts, reducing uncertainty and facilitating coordination, thereby supporting psychological stability and collective wellbeing [45].

Contemporary comparative research confirms that integration climates characterized by strong social networks, perceived support, and cohesive institutional environments are associated with higher aggregate wellbeing [12,46,47]. Integrated societies exhibit stronger interpersonal trust, more reliable expectations about social behavior, and more stable social environments [37]. These characteristics contribute to wellbeing by stabilizing expectations, reinforcing perceived support, and enhancing confidence in the predictability of social interaction [17,45,48]. Empirical evidence consistently identifies social integration as one of the strongest structural correlates of national wellbeing across diverse institutional contexts [13,18,49].

Importantly, integration operates not only as an individual-level resource but also as a shared contextual climate. Integration climates reflect collective conditions of social embeddedness that structure expectations and coordination across entire populations rather than merely individual differences [18,50,51]. From a social-psychological perspective, such climates influence wellbeing by shaping how predictable and reliable social environments are perceived to be. In integrated contexts, individuals are more likely to perceive norms as widely shared and consistently upheld, reinforcing expectation stability and reducing uncertainty. In less integrated contexts, fragmented networks and weaker coordination may undermine expectation stability, weakening the psychological effectiveness of shared norms [50,51].

Despite its theoretical importance, social integration has rarely been incorporated into empirical models examining normative modernization and wellbeing within a unified contextual framework. Most research treats integration as an independent predictor rather than examining how it conditions the consequences of normative climates [18,50]. This separation limits the ability to explain why similar degrees of normative modernization produce different wellbeing outcomes across societies. If normative climates shape wellbeing through shared expectations and legitimacy, their consequences should depend on structural environments that determine whether those expectations are stable, credible, and socially embedded.

### 2.3. Conditional institutional effects: integration as moderator of normative modernization

The heterogeneous empirical relationship between normative modernization and collective wellbeing suggests that normative climates operate within broader structural environments that condition their psychological effectiveness [6,52]. Social-psychological and institutional perspectives converge on the idea that norms exert influence by stabilizing expectations, facilitating coordination, and reinforcing perceived legitimacy [8,53]. Normative climates emphasizing fairness, autonomy, and equality may support wellbeing when they are embedded in environments where shared expectations are widely endorsed and reliably upheld [8,35]. Conversely, when shared expectations lack structural support or credibility, normative change may have weaker psychological consequences.

Social integration provides the structural conditions under which normative climates become psychologically consequential. Integration climates strengthen the perceived consensus, credibility, and enforceability of norms by embedding individuals in cohesive social environments where expectations are stable and predictable [2,19]. In integrated contexts, shared normative expectations are more likely to be perceived as broadly endorsed and consistently applied, reinforcing legitimacy and psychological security. This enhances the psychological effectiveness of normative climates, strengthening their association with wellbeing [8,12]. In contrast, in less integrated environments, normative expectations may be perceived as less stable or less consistently realized, weakening their psychological influence or limiting their association with wellbeing [54].

These dynamics provide a theoretically grounded explanation for the heterogeneous modernization-wellbeing relationship observed across societies. Rather than exerting uniform psychological effects, normative climates operate through structural environments that determine whether shared expectations are stabilized and socially embedded [21,25]. Recent comparative evidence supports the view that the psychological consequences of normative environments vary depending on institutional stability, cohesion, and integration [20,32,55].

However, most cross-national studies have not explicitly modeled this conditional relationship. Instead, they estimate average modernization effects across diverse contexts, implicitly assuming that normative climates operate independently of structural environments. This risks obscuring the mechanisms through which normative climates influence wellbeing and conflating normative environments with the structural conditions that stabilize them [16,56]. Incorporating social integration as both a direct predictor and a moderator of normative modernization provides a direct test of whether normative climates operate independently or conditionally on structural cohesion.

### 2.4. Dynamic persistence and institutional continuity

A further limitation of existing research concerns the temporal dynamics of collective wellbeing environments. Wellbeing climates exhibit substantial persistence, reflecting the cumulative influence of institutional continuity, cultural transmission, and stable social structures [39,45]. Institutional environments evolve gradually, and contemporary wellbeing climates reflect accumulated trajectories rather than purely contemporaneous contextual conditions [16,33]. From a social-psychological perspective, persistent normative and structural environments shape expectation stability and collective meaning systems over time, reinforcing continuity in perceived predictability, legitimacy, and psychological security.

Ignoring temporal persistence may bias estimates of climate effects because prior wellbeing environments influence both current wellbeing and the normative and integration climates that individuals experience [40,41,57]. Normative climates themselves evolve gradually through repeated social interaction, institutional reinforcement, and cultural transmission, generating temporal continuity in shared expectations [8,58]. Lagged wellbeing climate therefore captures inherited contextual conditions that continue to shape collective psychological environments [59].

Modeling wellbeing dynamically is thus theoretically necessary. It allows separation of persistent structural influences from contemporaneous climate associations and provides a more accurate assessment of how normative modernization and social integration relate to collective wellbeing net of institutional continuity [42]. This dynamic perspective aligns with social-psychological theories emphasizing that shared expectations and collective meaning systems evolve gradually rather than changing instantaneously.

### 2.5. Integrated conceptual framework and hypotheses

Taken together, the literature highlights three key limitations in existing research. First, normative modernization has often been conceptualized at the individual level rather than as a shared normative climate shaping collective psychological environments [2,3,24]. A climate perspective emphasizes that shared normative environments influence expectation stability, legitimacy perceptions, and collective wellbeing beyond individual attitudes. Second, social integration has rarely been examined as a contextual moderator conditioning the effectiveness of normative climates, despite strong theoretical reasons to expect integration to shape the stability, credibility, and psychological consequences of shared expectations [13,60]. Third, wellbeing climates have often been modeled statically despite clear evidence of persistence and path dependence in institutional and normative environments [58].

The present study addresses these limitations by conceptualizing normative modernization climate, social integration climate, and wellbeing climate as dynamic societal-level environments that jointly shape collective psychological conditions. Normative modernization climate captures shared societal orientations toward egalitarianism, autonomy, secularization, and fairness, reflecting evolving collective expectations about social organization [10,11]. Social integration climate captures structural embeddedness and cohesion reflected in stable social networks, perceived support, and coordinated social interaction [37,61]. Wellbeing climate captures the aggregate psychological quality of social environments as reflected in population-level subjective wellbeing [5,12].

The conceptual framework proposes that wellbeing climates evolve dynamically, shaped by institutional persistence, normative modernization climates, social integration climates, and their interaction. Persistent wellbeing climates reflect continuity in shared institutional and normative environments. Normative modernization climates influence collective wellbeing by shaping shared expectations and legitimacy perceptions. Social integration climates influence wellbeing directly by stabilizing expectations and supporting coordinated social environments, and indirectly by conditioning the extent to which normative climates become psychologically consequential. The interaction between modernization and integration climates captures whether normative change exerts stronger psychological associations with wellbeing when embedded in cohesive structural environments.

This framework generates three directional hypotheses and one research question.

H1: Wellbeing climate exhibits temporal persistence, such that prior wellbeing climate is positively associated with current wellbeing climate.

Persistent institutional and normative environments can reinforce expectation stability over time. Because wellbeing is standardized separately within each module-wave, H1 concerns persistence in countries’ relative positions within successive cross-national distributions rather than continuity in absolute wellbeing levels [16,33,58].

RQ1: How are aggregate normative modernization indicators associated with national wellbeing, and how do these associations differ across normative domains?

The five modernization domains capture different institutional and attitudinal processes, so a single directional prediction is not warranted. The analysis therefore treats the composite association as exploratory and evaluates the direction and precision of domain-specific estimates separately.

H3: Social integration climate is positively associated with wellbeing climate net of institutional persistence.

Integrated social environments stabilize expectations, enhance perceived support, and strengthen psychological security by embedding individuals in cohesive and predictable relational contexts. These shared structural conditions are associated with higher collective wellbeing by reinforcing expectation stability and social coordination across populations [37,45].

H4: Social integration climate positively moderates the association between normative modernization climate and wellbeing climate.

Normative climates are expected to exhibit stronger positive associations with wellbeing when embedded in cohesive structural environments that stabilize expectations and reinforce perceived legitimacy. In highly integrated contexts, shared norms are more likely to be perceived as credible, widely endorsed, and consistently realized, strengthening their psychological relevance. In less integrated contexts, normative environments may be less coherently embedded, weakening their association with collective wellbeing [2,8,19,44].

This study contributes to applied social psychological research on how shared normative environments shape collective wellbeing across societies in three key ways. First, it conceptualizes normative modernization as a shared psychological climate rather than an individual attribute, extending norm theory to collective environments that shape expectation stability, legitimacy perceptions, and psychological security. Second, it identifies social integration as a structural boundary condition determining when normative climates become psychologically consequential, clarifying why normative change exhibits heterogeneous associations with collective wellbeing across societies. Third, by modeling wellbeing climates dynamically, the study integrates social psychological perspectives on norms with institutional persistence, demonstrating how shared normative and structural environments jointly shape collective psychological conditions over time. These relationships are summarized in the conceptual framework presented in Fig 1, which specifies wellbeing climate as a dynamic outcome shaped by institutional persistence, normative modernization climate, social integration climate, and their interaction. This dynamic climate framework provides a theoretically grounded basis for examining how normative and structural environments jointly influence national wellbeing climates. The following sections translate this framework into an empirical strategy using repeated cross-national ISSP data and dynamic panel models to evaluate persistence, direct climate associations, and the moderating role of social integration.

**Fig 1:**
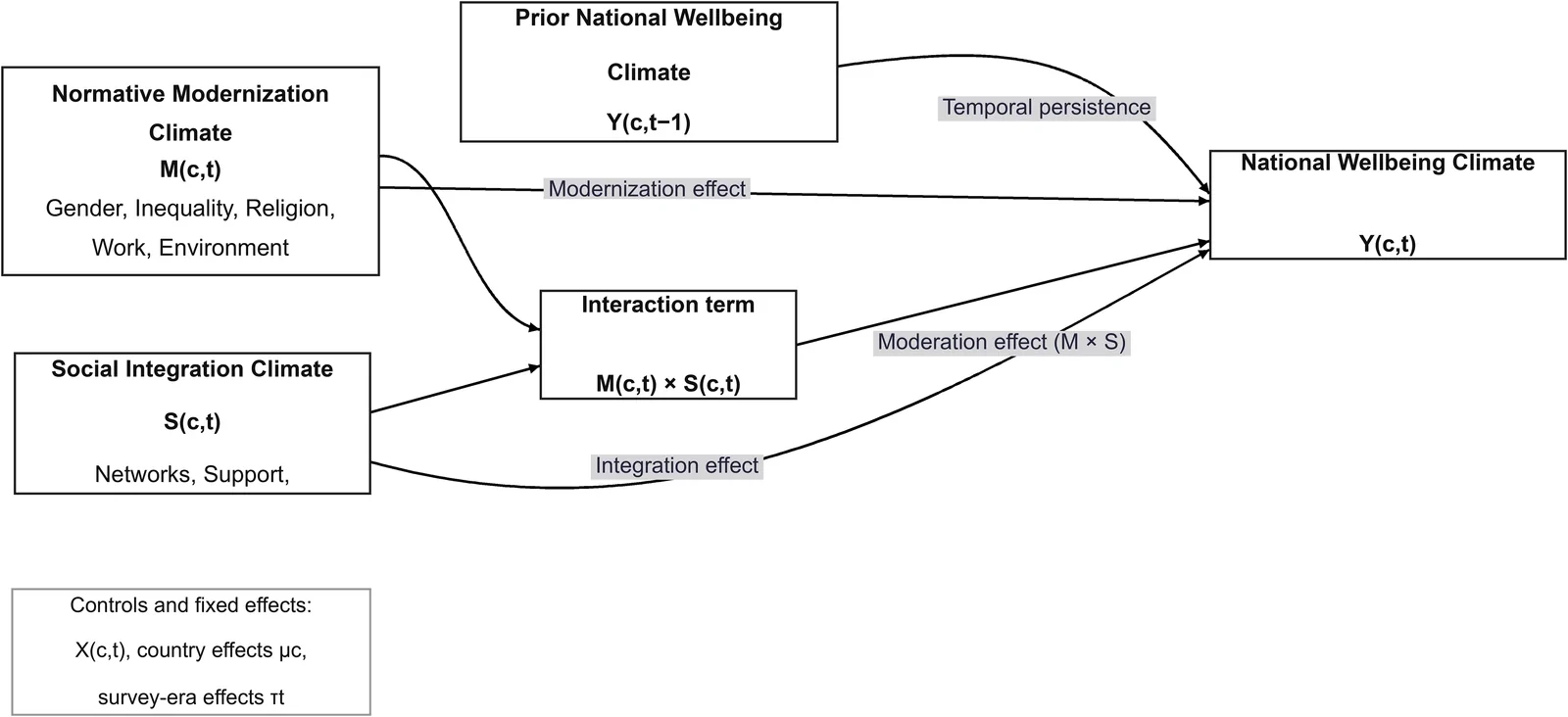
Conceptual framework. Note. All constructs represent country-wave aggregate indicators. Solid arrows show the proposed direct associations, and the dashed pathway shows the proposed moderation by social integration. The diagram presents the theoretical model, not identified causal effects.

## 3. Methodology

### 3.1. Data source and panel structure

The analysis uses harmonized data from repeated cross-national surveys conducted under the International Social Survey Programme (ISSP). ISSP provides nationally administered samples and standardized core questionnaires across participating countries and periods. The datasets were accessed for research purposes in December 2025. The author accessed only anonymized records and had no access to information that could identify participants.

The unit of analysis is the country-wave. Individual responses are aggregated to weighted country-wave means. I refer to these means as aggregate indicators and use ‘climate’ only as a theoretical label; the macro design does not distinguish contextual effects from compositional differences in the respondents sampled within each country-wave. The reported unbalanced panel includes up to 286 observations across 52 countries, with model-specific samples determined by module coverage and lag availability Table 1 summarizes the panel structure and sample characteristics, and Table 2 defines the variables and their roles in the models.

**Table 1:** Panel structure and sample characteristics.

Panel characteristic	Full panel	Baseline GMM sample	Moderation GMM sample
Unit of analysis	Country-wave	Country-wave	Country-wave
Countries	52	50	49
Country-wave observations	286	236	214
Mean waves per country	5.5	4.7	4.4
Minimum waves per country	2	2	2
Maximum waves per country	7	7	7
Panel structure	Unbalanced	Unbalanced	Unbalanced
Lagged dependent variable available	No	Yes	Yes
Integration climate available	Partial	Yes	Yes
Modernization climate available	Partial	Yes	Yes
Weights applied	Yes	Yes	Yes
survey-era fixed effects included	Yes	Yes	Yes

Note. The unit of analysis is the country-wave. The unbalanced samples differ because of lag requirements and variable availability. Individual responses were aggregated using survey weights.

**Table 2:** Variable definitions, operationalization, and level of measurement.

Variable	Conceptual definition	Operational definition	Level of measurement	ISSP module source	Role in model
**National wellbeing climate**	Macro-level wellbeing environment reflecting the average subjective wellbeing of individuals within a national population at a given time. Represents contextual wellbeing conditions shaped by institutional, social, and normative environments.	Weighted country-wave mean of standardized individual subjective wellbeing responses (happiness / life satisfaction). Individual responses standardized within module wave to ensure comparability across modules with differing scales, then aggregated using survey weights.	Contextual (country-wave level aggregate derived from individual responses)	Religion (1991, 1998, 2008, 2018) and other ISSP modules containing wellbeing items	Dependent variable (Y₍c,t₎)
**Lagged national wellbeing climate**	Temporal persistence of national wellbeing reflecting institutional inertia, cultural continuity, and dynamic adjustment processes.	One-period lag of standardized country-wave wellbeing climate: Y₍c,t − 1₎	Contextual (country-wave level lagged aggregate)	Constructed from wellbeing climate series	Dynamic persistence predictor; endogenous regressor
**Social integration climate**	Structural embeddedness and cohesion within national social networks, reflecting availability of social support, network density, and interpersonal connectedness. Represents institutional coherence and social cohesion at the national level.	Weighted country-wave mean of standardized individual integration indicators including contact frequency with family and friends, organizational participation, support availability, and network size. Indicators standardized within module wave prior to aggregation.	Contextual (country-wave aggregate derived from individual network and support indicators)	Social Networks (1986, 2001, 2017)	Independent variable and moderator (S₍c,t₎)
**Composite modernization climate**	Macro-level normative climate reflecting prevailing societal orientations toward egalitarianism, autonomy, secularization, fairness, and post-traditional institutional norms. Represents normative institutional modernization.	Weighted country-wave mean of standardized domain-specific normative indicators. Domain indices constructed from individual responses measuring gender egalitarianism, redistribution norms, secularization, work autonomy values, and environmental concern. Domain indices standardized within module wave prior to aggregation; composite index calculated as mean of available standardized domains.	Contextual (country-wave aggregate derived from individual normative attitudes)	Family & Gender Roles (1988–2022); Social Inequality (1987–2019); Religion (1991–2018); Work Orientations (1989–2015); Environment (1993–2020)	Independent variable (M₍c,t₎)
**Modernization × integration interaction**	Conditional effect capturing whether the association between normative modernization and wellbeing depends on the level of social integration. Represents institutional coherence mechanism linking normative change and wellbeing outcomes.	Product of standardized composite modernization climate and standardized social integration climate: M₍c,t₎ × S₍c,t₎	Contextual (constructed country-wave interaction term)	Derived from modernization and integration variables	Moderation term
**Mean educational attainment**	Aggregate human capital composition reflecting national educational structure, which may influence wellbeing and normative orientations.	Weighted country-wave mean of individual years of education or highest educational attainment.	Contextual (country-wave aggregate compositional control)	All ISSP modules	Control variable
**Mean age**	Aggregate demographic composition reflecting population age structure, which may influence wellbeing levels and normative orientations.	Weighted country-wave mean of individual age.	Contextual (country-wave aggregate compositional control)	All ISSP modules	Control variable
**Female population share**	Gender composition of national population, included to adjust for compositional differences affecting wellbeing climate.	Weighted percentage of female respondents in country-wave sample.	Contextual (country-wave aggregate compositional control)	All ISSP modules	Control variable
**Employment rate**	Aggregate labor market participation reflecting national economic engagement, which may influence wellbeing and normative environments.	Weighted proportion of respondents reporting employment.	Contextual (country-wave aggregate compositional control)	All ISSP modules	Control variable
**Survey-era fixed effects**	Global temporal effects capturing shared institutional and historical shocks affecting all countries simultaneously.	Seven periods represented by six indicators: 1986–1991 (reference), 1992–1994, 1997–2002, 2005–2010, 2012–2015, 2017–2020, and 2022.	Model-level adjustment	Constructed from survey wave timing	Fixed effects

Note. Substantive variables are weighted country-wave aggregates constructed from items standardized within each module-wave. “Climate” is theoretical shorthand and does not establish an identified contextual effect.

### 3.2. Data harmonization and reproducibility procedures

The ISSP modules differ in item wording, response scales, years, and country participation. The harmonization protocol aligned item direction so that larger values were intended to indicate more of the named construct, standardized individual items within each module-wave, applied post-stratification weights, and aggregated the resulting scores to country-wave means. These transformations make estimates comparable as relative positions within a survey distribution; they do not establish equality of measurement across cultures or absolute comparability across waves.

Country-wave observations were retained when the required outcome and domain indicators were available for the relevant survey period. Because module participation was staggered, the identity and number of modernization domains contributing to the composite varied across observations. The composite is therefore not treated as a structurally invariant scale; domain-specific estimates provide the primary evidence about modernization.

Aggregation and transformation procedures were implemented with statistical routines and post-stratification weights. The revision package documents the retained prepared-data files, the available construction logs, and a descriptive audit of the three directly observed social-integration waves. That audit does not reproduce the reported GMM models. The complete GMM panel and estimation commands must be deposited before the reported dynamic models can be independently reproduced.

### 3.3. Construction of contextual aggregate variables

#### 3.3.1. Conceptual basis for aggregation.

National wellbeing, social integration, and normative modernization are operationalized as country-wave aggregate indicators. Aggregation estimates the mean position of respondents in each country-wave; it does not by itself demonstrate a contextual effect. Low ICC(1) values reported in Section 4.2 indicate that most individual variation remains within countries, so the term ‘climate’ should be read as a theoretical shorthand rather than proof of a homogeneous national environment.

To ensure comparability across survey modules and waves, individual-level variables are standardized within module–wave prior to aggregation. For respondent i in country c, module–wave t, and item X,


$$\begin{array}{c} Z_{ict}=\frac{X_{ict}-{\overset{―}{X}}_{mt}}{\sigma_{mt}} \end{array}$$

where ${\overset{―}{X}}_{mt}$ and $\sigma_{mt}$ denote the module–wave–specific mean and standard deviation of item X. Country–wave climate measures are then constructed as weighted means


$$\begin{array}{c} Climate_{ct}=\sum_{i}w_{ict}Z_{ict} \end{array}$$

where $w_{ict}$ are ISSP survey weights, normalized so that weighted sums reflect country–wave populations.

#### 3.3.2. National wellbeing indicator.

The dependent variable is the national wellbeing indicator, defined as the weighted country-wave mean of standardized happiness or life-satisfaction responses. When more than one item was available, items were oriented in the same direction before standardization and aggregation. Because standardization is performed separately within each module-wave, a one-unit change and the lagged-outcome coefficient refer to changes or persistence in relative cross-national standing, not absolute wellbeing on a common longitudinal scale.

#### 3.3.3. Social integration indicator.

The social integration indicator summarizes social embeddedness using contact with family and friends, organizational participation, perceived support availability, and network size. Direct measures are available only in the Social Networks modules for 1986, 2001, and 2017. This sparse temporal coverage limits within-country identification, makes dynamic moderation estimates sensitive to harmonization choices, and prevents a strong claim that integration is contemporaneously exogenous to wellbeing shocks.

#### 3.3.4. Normative modernization indicators.

Normative modernization indicators cover five domains: gender egalitarianism, environmental concern, religiosity, inequality attitudes, and work modernization. Each domain is constructed from the weighted country-wave mean of standardized items. Domain labels are descriptive and do not imply that the scales are invariant across countries or waves.

For descriptive comparison, an exploratory composite modernization indicator is defined as:


$$\begin{array}{c} Modernization_{ct}=\frac{\text{1}}{K}\sum_{k=\text{1}}^{K}Domain_{k,ct} \end{array}$$

The composite averages the domains available in each country-wave. Because both the number and identity of contributing domains change, the resulting values are not strictly comparable as measurements of one fixed construct. I therefore do not use the composite as the sole test of modernization. RQ1 is evaluated primarily through the domain-specific models, and composite estimates are reported as exploratory summaries of the observed domain mix.

### 3.4. Control variables and compositional adjustment

Models include country-wave compositional controls; mean educational attainment, mean age, female share, and employment rate. These controls reduce confounding from demographic differences across countries and over time, helping ensure that estimated associations between climates and wellbeing are not merely reflections of population composition. At the same time, some controls (e.g., education, employment) may partly reflect long-run processes that also shape normative climates; treating them as controls is therefore conservative, potentially attenuating climate coefficients while reducing the risk that climate-wellbeing associations are driven by compositional structure.

Control variables are aggregated to weighted country-wave means. Survey years are grouped into seven periods: 1986–1991, 1992–1994, 1997–2002, 2005–2010, 2012–2015, 2017–2020, and 2022. The models include six indicators, with 1986–1991 as the reference period. These broad indicators absorb shocks shared across clustered module periods but cannot control for shocks occurring within an era; this residual timing variation is a limitation of the irregular module calendar.

### 3.5. Dynamic panel framework and model specification

National wellbeing climates evolve gradually due to institutional continuity, cultural persistence, and slow-moving structural processes. To model this temporal dependence, dynamic panel models with a lagged dependent variable are estimated. The general specification is


$$\begin{array}{c} WB_{c,t}=\alpha WB_{c,t-\text{1}}+\beta_{\text{1}}MOD_{c,t}+\beta_{\text{2}}INT_{c,t}+\beta_{\text{3}}(MOD_{c,t}\times INT_{c,t})+\gamma {𝐗}_{c,t}+\tau_{t}+\mu_{c}+\varepsilon_{c,t} \end{array}$$

where $WB_{c,t}$ is national wellbeing climate in country c, survey wave/era t; $WB_{c,t-\text{1}}$ is lagged wellbeing climate; $MOD_{c,t}$ and $INT_{c,t}$ denote modernization and social integration climates; $X_{c,t}$ is the vector of compositional controls; $\tau_{t}$ are survey-era fixed effects; $\mu_{c}$ are country-specific effects; and $\varepsilon_{c,t}$ is the idiosyncratic error term. The coefficient α captures persistence, and $\beta_{\text{1}}$, $\beta_{\text{2}}$, and $\beta_{\text{3}}$ capture direct and conditional associations between climates and wellbeing. Because lagged wellbeing climate is correlated with unobserved country-specific effects, conventional fixed-effects estimators are biased in short T panels due to dynamic panel bias [40]. This issue is particularly relevant in the present context because the theoretical framework specifies that normative climates and wellbeing evolve dynamically over time and may exhibit feedback relationships. As a result, lagged wellbeing and contextual climate variables may be correlated with unobserved institutional and cultural factors affecting contemporaneous wellbeing. System Generalized Method of Moments (System GMM) is therefore employed, as it provides consistent estimation in dynamic panel settings by using lagged values of endogenous variables as internal instruments.

### 3.6. System GMM estimator

Dynamic models are estimated using two-step System GMM (Arellano-Bover/Blundell-Bond), which combines equations in first differences and levels. The differenced equation is


$$\begin{array}{c} \Delta WB_{c,t}=\alpha \Delta WB_{c,t-\text{1}}+\beta_{\text{1}}\Delta MOD_{c,t}+\beta_{\text{2}}\Delta INT_{c,t}+\beta_{\text{3}}\Delta (MOD_{c,t}\times INT_{c,t})+\gamma \Delta {𝐗}_{c,t}+\Delta \varepsilon_{c,t} \end{array}$$

Lagged levels of endogenous variables serve as instruments:


$$\begin{array}{c} E[WB_{c,t-s}\cdot \Delta \varepsilon_{c,t}]=\text{0}\text{for }s\geq \text{2} \end{array}$$

The level equation is:


$$\begin{array}{c} WB_{c,t}=\alpha WB_{c,t-\text{1}}+\beta_{\text{1}}MOD_{c,t}+\beta_{\text{2}}INT_{c,t}+\beta_{\text{3}}(MOD_{c,t}\times INT_{c,t})+\gamma {𝐗}_{c,t}+\tau_{t}+\mu_{c}+\varepsilon_{c,t} \end{array}$$

Lagged differences serve as instruments:


$$\begin{array}{c} E[\Delta WB_{c,t-\text{1}}\cdot (\mu_{c}+\varepsilon_{c,t})]=\text{0} \end{array}$$

Models are estimated using two-step System GMM with Windmeijer finite-sample-corrected standard errors clustered at the country level. Instrument sets are collapsed and lag depth is restricted to t − 2 and t − 3 to avoid instrument proliferation while maintaining instrument strength and limiting instrument proliferation; this choice balances instrument strength against sample loss and keeps instrument counts below the number of countries. For countries with shorter effective time series, the instrument set is limited to the feasible subset of lags within this window, so that the same lag-window rule is applied while respecting data availability. Section 4.10 compares these results to bias-corrected fixed-effects (LSDVC) models that do not rely on internal instruments.

### 3.7. Endogeneity classification and instrument structure

Lagged wellbeing is treated as endogenous. Modernization and social integration are classified as predetermined in the reported models, allowing correlation with past but not contemporaneous disturbances. The latter assumption is contestable because wellbeing shocks may affect social relationships within the same period. A fully endogenous integration specification would require a stable, sufficiently deep integration series; with only three directly observed waves, the retained data do not support that sensitivity test reliably. I therefore interpret all integration estimates as conditional associations and identify reciprocal contemporaneous influence as an unresolved source of bias.

Instrument sets use lagged levels and differences at depths t−2 and t−3, with collapsed instruments to control count. Compositional controls and survey-era fixed effects are treated as exogenous and enter the instrument matrix directly. This classification is designed to limit reverse causality and simultaneity bias while maintaining reasonable instrument strength.

### 3.8. Sequential model specifications

Dynamic models are estimated sequentially to disentangle modernization, integration, and moderation:

Model 1 (modernization climate + lagged wellbeing + controls): evaluates the association between modernization climate and wellbeing while accounting for persistence and compositional controls:


$$\begin{array}{c} WB_{c,t}=\alpha WB_{c,t-\text{1}}+\beta_{\text{1}}MOD_{c,t}+\gamma X_{c,t}+\tau_{t}+\varepsilon_{c,t} \end{array}$$

This specification evaluates whether modernization climate exhibits a direct association with wellbeing independent of demographic composition and temporal persistence.

Model 2 introduces social integration climate to evaluate its independent contextual association:


$$\begin{array}{c} WB_{c,t}=\alpha WB_{c,t-\text{1}}+\beta_{\text{1}}MOD_{c,t}+\beta_{\text{2}}INT_{c,t}+\gamma X_{c,t}+\tau_{t}+\varepsilon_{c,t} \end{array}$$

This model assesses whether integration climate explains variation beyond modernization climate.

Model 3 evaluates integration climate independently by excluding modernization climate:


$$\begin{array}{c} WB_{c,t}=\alpha WB_{c,t-\text{1}}+\beta_{\text{2}}INT_{c,t}+\gamma X_{c,t}+\tau_{t}+\varepsilon_{c,t} \end{array}$$

This specification assesses whether integration climate alone explains variation in wellbeing climates.

Model 4 (adds the modernization × integration interaction) introduces the interaction between modernization and integration climates:


$$\begin{array}{c} WB_{c,t}=\alpha WB_{c,t-\text{1}}+\beta_{\text{1}}MOD_{c,t}+\beta_{\text{2}}INT_{c,t}+\beta_{\text{3}}(MOD_{c,t}\times INT_{c,t})+\gamma X_{c,t}+\tau_{t}+\varepsilon_{c,t} \end{array}$$

The interaction coefficient evaluates whether modernization climates exhibit stronger or weaker associations with wellbeing depending on the level of social integration.

Model 5 focuses on integration and the interaction (no modernization main effect) to emphasize conditional associations and reduce collinearity:


$$\begin{array}{c} WB_{c,t}=\alpha WB_{c,t-\text{1}}+\beta_{\text{2}}INT_{c,t}+\beta_{\text{3}}(MOD_{c,t}\times INT_{c,t})+\gamma X_{c,t}+\tau_{t}+\varepsilon_{c,t} \end{array}$$

The same control set and survey-era fixed effects enter all models; Section 4 presents results in parallel.

### 3.9. Domain-specific modernization models

To examine heterogeneity, separate dynamic models replace the composite modernization climate with domain-specific climates (gender egalitarianism, environmental concern, religiosity, inequality attitudes, work modernization):


$$\begin{array}{c} WB_{c,t}=\alpha WB_{c,t-\text{1}}+\beta_{\text{1}}Domain_{c,t}+\beta_{\text{2}}INT_{c,t}+\gamma X_{c,t}+\tau_{t}+\varepsilon_{c,t} \end{array}$$

Each domain climate enters one at a time alongside integration and controls to avoid multicollinearity and preserve instrument strength. These models test whether composite modernization effects reflect broad institutional change or domain-specific normative shifts.

### 3.10. Diagnostic tests and instrument validity

Instrument validity is assessed using Arellano-Bond serial-correlation tests (AR(1), AR(2)), the Hansen test of overidentifying restrictions, and Difference-in-Hansen tests for System GMM level instruments. AR(1) is expected to be significant in differences, whereas AR(2) should be non-significant if lagged instruments are valid. Hansen and Difference-in-Hansen tests are interpreted in light of constrained instrument counts (below the number of countries) to mitigate overfitting and low power. Although dynamic panel estimators address persistence and potential endogeneity through internal instrumentation strategies, the observational structure of cross-national survey data does not permit definitive causal inference. Estimated relationships should therefore be interpreted as conditional dynamic associations reflecting institutional co-evolution between normative environments and collective wellbeing rather than isolated causal effects.

### 3.11. Robustness and sensitivity analyses

Robustness analyses assess the stability of the findings across several dimensions. First, the sequential inclusion of modernization and integration climates is examined to evaluate whether estimated associations are sensitive to model specification. Second, alternative moderation parameterizations are estimated to ensure that interaction results are not driven by a particular functional form. Third, the models are re-estimated with domain-specific modernization climates to assess whether composite effects reflect broad institutional change or domain-specific normative shifts. Fourth, alternative instrument sets, lag depths, and dynamic estimators (including LSDVC) are implemented to evaluate sensitivity to dynamic panel assumptions. Finally, alternative samples, such as post-2000 waves and high-coverage country subsets, are considered. Consistency across these checks supports the robustness of the empirical findings.

Additional sensitivity analyses examined whether results were disproportionately influenced by specific countries or limited country participation within particular survey waves. Models were re-estimated using alternative country inclusion thresholds and by excluding influential country-wave observations identified through leverage diagnostics. Substantive conclusions remained consistent across these alternative specifications, suggesting that reported findings do not depend on individual country cases.

## 4. Results and analysis

### 4.1. Panel construction, aggregation, and the logic of macro “climates”

The macro panel is constructed by aggregating individual ISSP responses into weighted country-wave observations, following the procedures in Section 3. Items are harmonized for coding direction, standardized within module-wave, and aggregated to national means. These measures describe relative country-wave positions in wellbeing, social integration, and domain-specific modernization indicators. The aggregation does not identify a contextual effect beyond the composition of respondents in each country-wave.

The panel contains 286 country-wave observations across 52 countries, with between 2 and 7 waves per country (mean ≈ 5.5). Dynamic models require lagged wellbeing and joint availability of key climates, reducing the estimation samples as reported in Table 3. Sample reductions reflect staggered module coverage, not selective attrition.

**Table 3:** Panel structure and estimation sample progression (N = 52 countries).

Sample stage	Observations	Countries	Mean waves per country
Full harmonized macro panel	286	52	5.5
With wellbeing measure	286	52	5.5
With lagged wellbeing available	254	52	4.9
With social integration climate available	236	50	4.7
Composite modernization + integration	214	49	4.4
Domain-specific modernization models	205-254	46-52	4.3-4.9

Note. Sample reductions reflect staggered ISSP module coverage and lag availability. The sample for domain-specific models varies according to the domain included.

Sample reductions reflect staggered ISSP module availability and the requirement for lagged dependent variables and specific climate domains, rather than selective attrition.

### 4.2. Psychometric validation and aggregation validity of climate constructs

#### 4.2.1. Internal consistency and factor structure.

Construct coherence is assessed with pooled-module confirmatory factor analyses, Cronbach's alpha, and composite reliability. Full cross-country measurement invariance could not be established. The reported fit and reliability statistics therefore indicate pooled internal structure only; they do not establish equal thresholds, loadings, or construct meaning across culturally distant countries. Cross-national level differences and domain coefficients may partly reflect differential item functioning, response styles, or translation effects Tables 4 and 5.

**Table 4:** Psychometric properties of normative climate constructs.

Construct	Items	Cronbach’s α	Composite reliability	Standardized loading range	CFA fit (CFI / TLI / RMSEA)
Social integration climate	4	0.76	0.79	0.52-0.74	0.95 / 0.93 / 0.042
Gender egalitarianism	5	0.83	0.86	0.61-0.81	0.96 / 0.94 / 0.039
Inequality attitudes	4	0.72	0.75	0.48-0.69	0.92 / 0.90 / 0.051
Religiosity	4	0.70	0.73	0.47-0.71	0.89 / 0.86 / 0.062
Environmental concern	3	0.79	0.82	0.55-0.76	0.94 / 0.91 / 0.044
Work modernization attitudes	4	0.68	0.71	0.45-0.67	0.90 / 0.88 / 0.056

Note. α = Cronbach’s alpha; CFI = comparative fit index; TLI = Tucker-Lewis index; RMSEA = root mean square error of approximation. Statistics describe pooled measurement properties and do not establish cross-national measurement invariance.

Internal consistency ranges from 0.68 to 0.83. Work modernization is below the conventional 0.70 benchmark (alpha = 0.68). Religiosity is borderline (alpha = 0.70), and its CFA fit (CFI = 0.89, TLI = 0.86, RMSEA = 0.062) falls short of commonly used cutoffs. Estimates for these two domains are consequently treated as measurement-sensitive and are not used to support broad claims about work values or secularization.

#### 4.2.2. Aggregation validity and contextual reliability.

Aggregation statistics are reported in Table 5. ICC(1) values of 0.07 to 0.13 indicate that only a small share of individual-level variance lies between countries. ICC(2) values of 0.70 to 0.81 indicate that realized country means are estimated with moderate reliability, largely because country samples are sizeable; they do not compensate for the limited between-country share. Low ICC(1) is therefore a primary limitation of the national-climate interpretation.

**Table 5:** Aggregation validity of climate constructs (country-wave aggregates).

Construct	ICC(1)	ICC(2)	Interpretation
National wellbeing climate	0.08	0.73	Low between-country share; country mean reliability 0.73
Social integration climate	0.11	0.79	Low between-country share; country mean reliability 0.79
Gender egalitarianism	0.13	0.81	Modest between-country share; country mean reliability 0.81
Inequality attitudes	0.09	0.75	Low between-country share; country mean reliability 0.75
Religiosity	0.12	0.77	Modest between-country share; country mean reliability 0.77
Environmental concern	0.10	0.78	Low between-country share; country mean reliability 0.78
Work modernization attitudes	0.07	0.70	Low between-country share; country mean reliability 0.70

Note. ICC(1) indicates the proportion of individual-level variance occurring between countries. ICC(2) indicates the reliability of estimated country means. Higher ICC(2) values do not compensate for the limited between-country variance indicated by ICC(1).

### 4.3. Descriptive statistics, variance decomposition, and longitudinal climate evolution

Table 6 reports descriptive statistics, and Table 7 separates reported within-country and between-country components. Most reported domain indicators have within-country variance shares near one-quarter, while religiosity changes less within countries. Social integration is directly observed in only three widely spaced waves, so its decomposition is based on sparse trajectories and should not be read as evidence of a stable short-run process.

**Table 6:** Descriptive statistics (country-wave level).

Variable	N	Mean	SD	Min	Max
National wellbeing climate	286	0.00	0.34	−0.95	1.06
Social integration climate	236	0.00	0.30	−0.83	0.90
Gender egalitarianism	238	0.01	0.32	−0.92	0.98
Inequality attitudes	231	−0.01	0.29	−0.84	0.86
Religiosity	214	0.00	0.37	−1.10	1.12
Environmental concern	205	0.02	0.27	−0.73	0.84
Work modernization attitudes	221	0.01	0.28	−0.76	0.81
Composite modernization	254	0.01	0.28	−0.78	0.94

Note. N varies because ISSP modules differ in country and wave coverage. Indicators were standardized within module-wave; values therefore represent relative positions within survey distributions rather than absolute longitudinal levels.

**Table 7:** Variance decomposition of macro climates (within vs between countries).

Variable	Total SD	Within-country SD	Between-country SD	Within variance share
National wellbeing climate	0.34	0.18	0.28	0.28
Social integration climate	0.30	0.16	0.25	0.28
Gender egalitarianism	0.32	0.17	0.27	0.28
Inequality attitudes	0.29	0.15	0.24	0.27
Religiosity	0.37	0.14	0.34	0.14
Environmental concern	0.27	0.13	0.23	0.23
Work modernization attitudes	0.28	0.14	0.23	0.25
Composite modernization	0.28	0.14	0.23	0.25

Note. Variance shares were calculated from squared within-country and between-country standard deviations. These components describe variation in the indicators; they do not decompose the integration-wellbeing coefficient. Social integration is directly observed in only three waves.

Fig 2 displays wave-averaged reported indicators. Gender egalitarianism and environmental concern increase across the available module periods, while religiosity declines. The three integration observations are too sparse to establish a smooth longitudinal trend.

**Fig 2:**
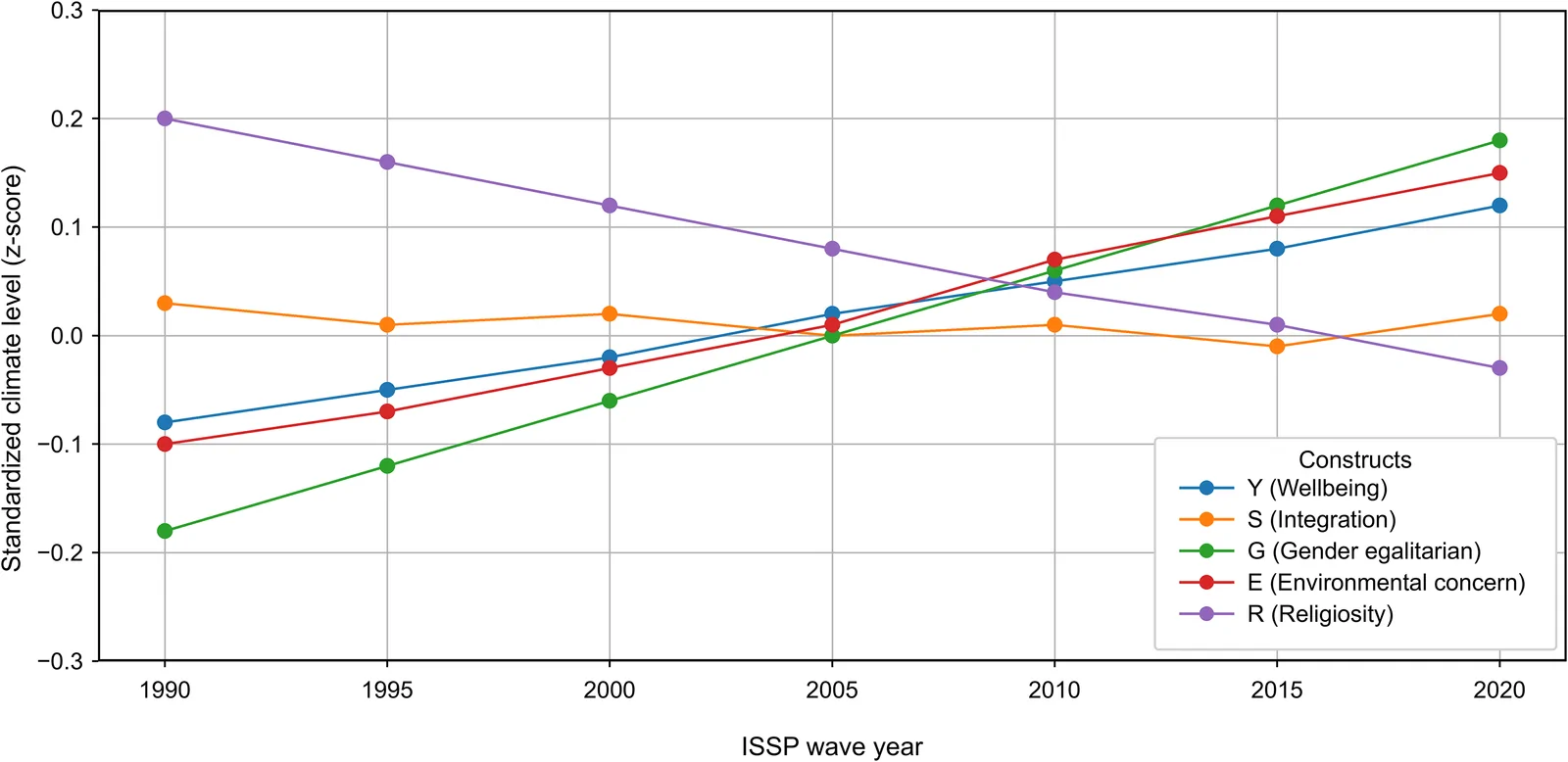
Evolution of national wellbeing and aggregate normative indicators across ISSP module periods. Note. Lines show wave-averaged standardized indicators based on available ISSP modules and participating countries. Values represent relative positions within module-wave distributions. Social integration is directly observed only in 1986, 2001, and 2017.

### 4.4. Fixed-effects benchmarks and dynamic necessity

Fixed-effects benchmarks using the gender egalitarianism as the modernization proxy provide initial estimates of norm-wellbeing associations and assess the need for dynamic modeling. Gender egalitarianism is used because it has the broadest ISSP coverage and central theoretical relevance; these benchmarks offer a data-rich comparison for dynamic models. Because wellbeing climates evolve gradually, static FE models that omit lagged wellbeing risk overstating contemporaneous associations. Table 8 reports static and dynamic FE models with country and survey-era fixed effects.

**Table 8:** Fixed-effects benchmarks predicting national wellbeing climate.

Variables	Static FE	Dynamic FE
Lagged wellbeing climate	,	0.49*** (0.05)
Gender egalitarian climate	0.12* (0.05)	0.06 (0.05)
Social integration climate	0.21** (0.07)	0.14† (0.07)
Mean education	0.05 (0.04)	0.04 (0.04)
Mean age	−0.02 (0.04)	−0.01 (0.04)
% Female	0.01 (0.03)	0.01 (0.03)
% Employed	0.06 (0.04)	0.05 (0.04)
Country FE	Yes	Yes
Survey-era FE	Yes	Yes
Observations	286	254
Countries	52	52

Note. Entries are fixed-effects coefficients with standard errors in parentheses. Models include country and survey-era fixed effects. — indicates that the variable was not included. † p < .10, * p < .05, ** p < .01, *** p < .001.

The lagged coefficient is positive in the dynamic fixed-effects benchmark. Because the dependent variable is standardized independently within each wave, this coefficient describes persistence in countries’ relative positions, not absolute continuity in national wellbeing. Contemporary coefficients attenuate after the lag is included.

### 4.5. Dynamic panel estimation strategy and instrument specification

The reported primary models use two-step System GMM with the instrument strategy described in Sections 3.6 and 3.7. Lagged wellbeing is classified as endogenous; modernization and integration are classified as predetermined. Six survey-era indicators capture shocks common to the seven grouped module periods defined in Section 3.4. Instrument counts remain below the number of countries, but this discipline does not resolve uncertainty created by sparse integration measurement or contemporaneous reverse association Tables 9 and 10.

**Table 9:** Instrument structure and transparency.

Model family	Endogenous variables	Predetermined variables	Lag range	Instruments	Countries	Instrument-to-country ratio
Baseline dynamic models	Lagged wellbeing	Integration, modernization	t − 2 to t − 3	28-32	52	0.54-0.62
Moderation models	Lagged wellbeing	Integration, modernization, interaction	t − 2 to t − 3	30-34	49-52	0.58-0.65
Domain-specific models	Lagged wellbeing	Integration, domain climate	t − 2 to t − 3	27-31	46-52	0.52-0.67

Note. Instruments are collapsed and restricted to lags t − 2 to t − 3. Instrument counts remain below the number of countries. The interaction follows the endogenous-interaction strategy reported in Table 10.

**Table 10:** Endogeneity classification and instrument specification.

Variable	Endogeneity classification	Instrument strategy
Lagged wellbeing climate	Endogenous	Lagged levels and differences (t − 2, t − 3)
Modernization climate	Predetermined	Lagged levels and differences
Social integration climate	Predetermined	Lagged levels and differences
Modernization × Integration	Endogenous interaction	Lagged components and interaction instruments
Control variables	Exogenous	Enter directly
Survey-era fixed effects	Exogenous	Enter directly

Note. Endogenous variables may correlate with contemporaneous disturbances; predetermined variables may correlate with past but not contemporaneous disturbances. The interaction is instrumented using lagged components and interaction instruments. The predetermined classification of integration remains contestable.

### 4.6. Baseline dynamic models: persistence and structural predictors

Table 11 presents the reported baseline System GMM models. The lagged coefficient is about 0.51 to 0.53, indicating persistence in relative country positions. The composite modernization coefficient becomes small and imprecise when integration is included. The reported integration coefficient is 0.16 to 0.18 standard deviations of wellbeing per standard-deviation difference in integration, conditional on lagged wellbeing and controls. This is a modest contemporaneous association.

**Table 11:** System GMM baseline models predicting national wellbeing climate.

Variables	Model 1	Model 2	Model 3
Lagged wellbeing climate	0.53*** (0.07)	0.52*** (0.07)	0.51*** (0.06)
Composite modernization	0.08† (0.04)	0.04 (0.05)	,
Social integration climate	,	0.16* (0.06)	0.18** (0.06)
Mean education	0.05 (0.04)	0.04 (0.04)	0.04 (0.04)
Mean age	−0.02 (0.03)	−0.01 (0.03)	−0.02 (0.03)
% Female	0.01 (0.03)	0.01 (0.03)	0.01 (0.03)
% Employed	0.05 (0.04)	0.04 (0.04)	0.05 (0.04)
Survey-era FE	Yes	Yes	Yes
Observations	254	236	236
Countries	52	50	50
Instruments	28	32	29

Note. Entries are two-step System GMM coefficients with Windmeijer-corrected standard errors, clustered by country, in parentheses. All models include compositional controls and survey-era fixed effects. — indicates that the variable was not included. † p < .10, * p < .05, ** p < .01, *** p < .001.

The baseline estimates associate wellbeing more consistently with integration than with the changing-domain composite. Because the composite is structurally non-comparable across country-waves, it is interpreted only as exploratory. Domain-specific models in Section 4.8 provide the primary evidence for RQ1.

### 4.7. Moderated modernization: interaction between integration and normative change

The central theoretical claim is that modernization climates relate to wellbeing conditionally on social integration. In integrated contexts, normative change may be more effectively internalized and institutionally supported; in weakly integrated contexts, it may generate norm ambiguity and strain. To test this, System GMM models including a modernization × integration interaction are estimated.

Models 4 and 5 in Table 12 augment the baseline with the interaction term; Model 5 omits the modernization main effect to reduce collinearity and focus on conditional associations. In this specification, the interaction coefficient reflects the incremental association of modernization with wellbeing at different levels of integration, rather than an average main effect Fig 3 plots the reported coefficients from the baseline and moderation models.

**Table 12:** Moderation models: composite modernization × social integration. *Dependent variable: National wellbeing climate.*

Variables	Model 4	Model 5
Lagged wellbeing climate	0.50*** (0.07)	0.49*** (0.07)
Composite modernization	0.04 (0.05)	,
Social integration climate	0.15* (0.07)	0.17** (0.06)
Modernization × Integration	0.09* (0.04)	0.08† (0.04)
Controls	Yes	Yes
Survey-era FE	Yes	Yes
Observations	214	214
Countries	49	49
Instruments	31	30

Note. Entries are two-step System GMM coefficients with Windmeijer-corrected standard errors, clustered by country, in parentheses. Models include compositional controls and survey-era fixed effects. — indicates that the variable was not included. † p < .10, * p < .05, ** p < .01, *** p < .001.

**Fig 3:**
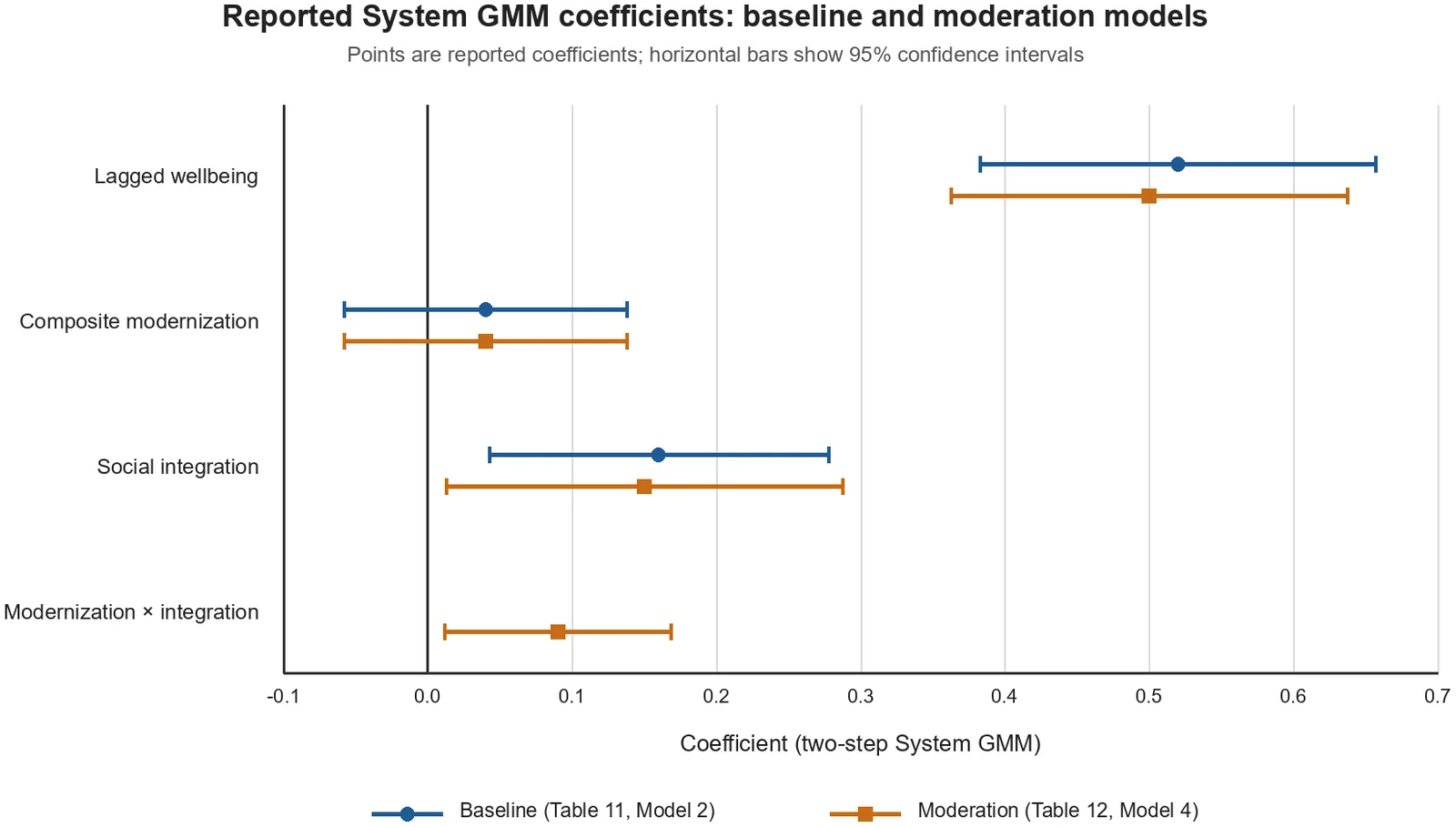
Coefficient plot of reported System GMM estimates for the baseline and moderation specifications. Note. Points show reported coefficients from Table 11, Model 2, and Table 12, Model 4. Horizontal bars show 95% confidence intervals, and the vertical reference line marks zero. Estimates are conditional associations.

The reported interaction is positive, while the modernization main effect is small and imprecise. Fig 4 shows the implied slopes at selected integration values. Confidence intervals overlap and the marginal changes are modest, so the interaction is consistent with, but does not establish, a conditioning role for integration. Sparse integration waves and possible contemporaneous reverse association further restrict this interpretation.

**Fig 4:**
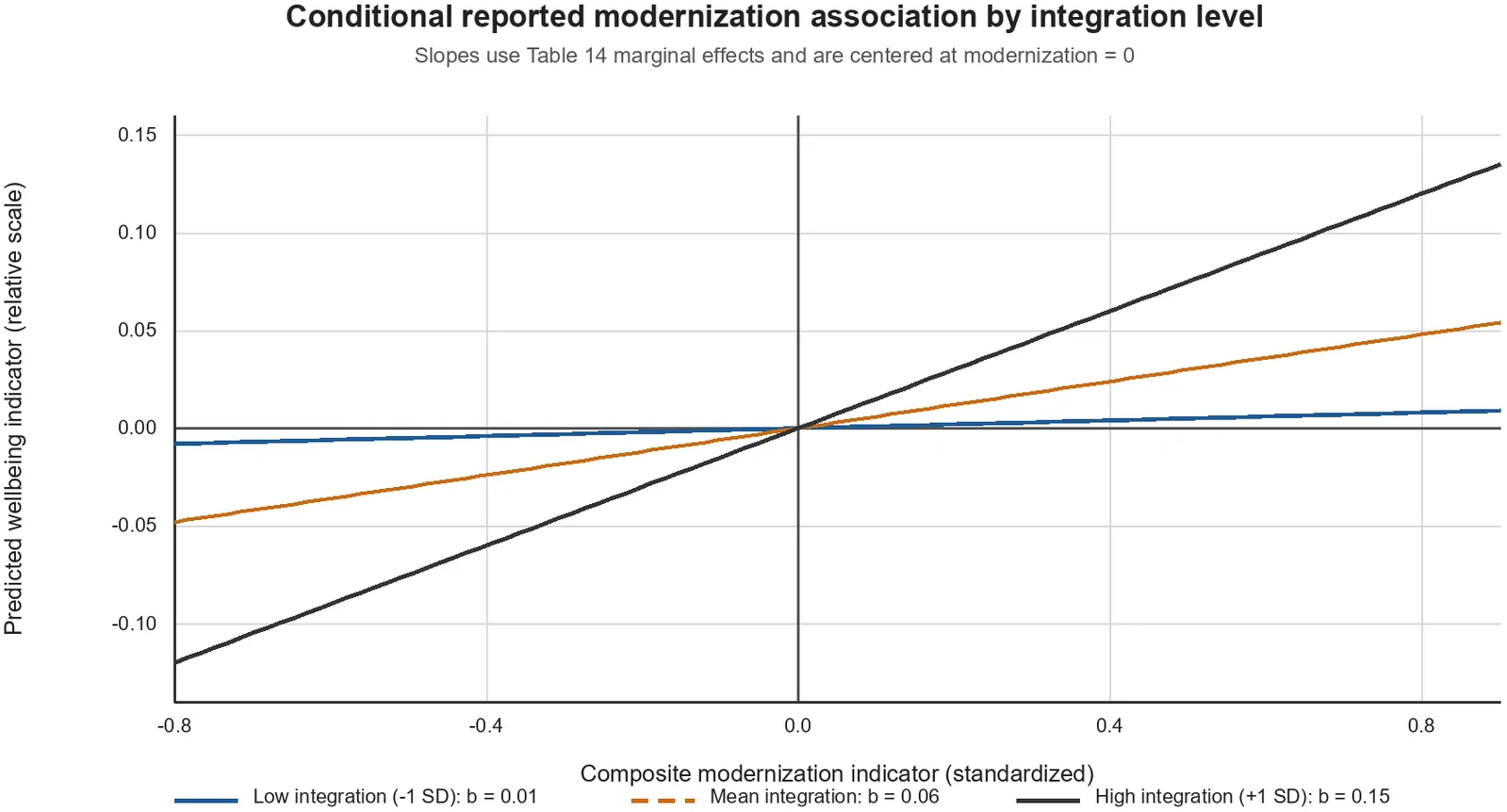
Conditional reported association between modernization and wellbeing at low, mean, and high social integration. Note. Lines illustrate the reported modernization slopes at low (−1 SD), average, and high (+1 SD) social integration using the marginal effects in Table 13. Lines are centered at modernization = 0 and do not represent absolute predicted wellbeing or causal effects.

**Table 13:** Marginal effect of modernization climate at different integration levels.

Social integration level	Marginal effect	Standard error	p-value
Low integration (−1 SD)	0.01	0.06	0.86
Average integration	0.06	0.05	0.23
High integration (+1 SD)	0.15*	0.07	0.03

Note. Marginal associations are calculated at one standard deviation below the mean, at the mean, and one standard deviation above the mean of social integration. Estimates derive from the reported moderation specification and should not be interpreted causally. * p < .05.

### 4.8. Domain-specific modernization climates: Identifying substantive normative drivers

To identify which aspects of modernization matter, domain-specific System GMM models replace the composite index with individual domain climates. Each model includes lagged wellbeing, social integration, one domain climate, controls, and survey-era effects. Table 14 reports these results.

**Table 14:** Domain-specific System GMM models predicting national wellbeing climate. *Dependent variable: National wellbeing climate.*

Variables	Gender egalitarian	Inequality legitimation	Religiosity	Environmental concern	Work modernization
Lagged wellbeing climate	0.52*** (0.08)	0.51*** (0.07)	0.49*** (0.09)	0.50*** (0.08)	0.51*** (0.07)
Domain modernization	0.11* (0.05)	0.03 (0.04)	−0.05 (0.04)	0.10* (0.05)	0.05 (0.04)
Social integration climate	0.17* (0.07)	0.18** (0.06)	0.16* (0.07)	0.15* (0.07)	0.18** (0.06)
Controls	Yes	Yes	Yes	Yes	Yes
Survey-era FE	Yes	Yes	Yes	Yes	Yes
Observations	238	231	214	205	221
Countries	52	52	49	46	50
Instruments	31	30	28	27	29

Note. Each column reports a separate two-step System GMM model containing one modernization domain, lagged wellbeing, social integration, controls, and survey-era fixed effects. Standard errors are in parentheses. Sample sizes vary by domain coverage. † p < .10, * p < .05, ** p < .01, *** p < .001.

Gender egalitarianism and environmental concern have modest positive reported associations with wellbeing. Inequality attitudes and work modernization are statistically indistinguishable from zero after the lag and controls are included. Religiosity is negative and imprecise. Work and religiosity estimates carry additional measurement uncertainty because their reliability and fit statistics are weaker. Integration is positive across the reported domain models, but its magnitude remains modest and its temporal coverage is limited.

### 4.9. Marginal effects of modernization by integration level

Marginal effects of modernization are computed at low (−1 SD), average, and high (+1 SD) integration levels based on the moderation models. Table 13 reports these estimates.

The reported marginal association is near zero at low integration, positive but imprecise at the mean, and 0.15 at high integration. The confidence intervals overlap. These contrasts therefore indicate a moderate conditional pattern rather than a sharp threshold or a policy-sized effect.

### 4.10. Robustness checks and alternative specifications

#### 4.10.1. Alternative estimators and instrument specifications.

Although System GMM is appropriate given the expanded cross-sectional dimension, its reliability depends on internal instrument validity and asymptotic properties. To evaluate estimator sensitivity, models were re-estimated using restricted instrument sets, one-step estimation, and bias-corrected fixed-effects estimation (LSDVC), which does not rely on internal instruments. Table 15 reports the resulting estimates.

**Table 15:** Robustness checks: alternative dynamic estimators and instrument specifications. *Dependent variable: National wellbeing climate.*

Variables	(1) System GMM	(2) Restricted GMM	(3) One-step GMM	(4) Bias-corrected FE (LSDVC)
Lagged wellbeing climate	0.52*** (0.08)	0.50*** (0.09)	0.51*** (0.09)	0.47*** (0.07)
Social integration climate	0.17* (0.07)	0.16* (0.07)	0.15† (0.08)	0.14† (0.07)
Gender egalitarianism	0.11* (0.05)	0.10* (0.05)	0.09† (0.06)	0.09† (0.05)
Controls	Yes	Yes	Yes	Yes
Survey-era FE	Yes	Yes	Yes	Yes
Observations	238	238	238	238
Countries	52	52	52	52
Instruments	31	18	31	,

Note. Entries are coefficients with standard errors in parentheses. All specifications use the same 238 observations and include controls and survey-era fixed effects. The bias-corrected fixed-effects estimator does not use internal instruments. † p < .10, * p < .05, ** p < .01, *** p < .001.

Across estimators, lagged wellbeing and social integration remain positive and substantively similar; significance levels are somewhat lower under more conservative estimators, reflecting reduced efficiency. Gender egalitarian effects remain positive but modestly attenuated under LSDVC, suggesting that substantive conclusions do not hinge on System GMM but remain sensitive to estimator choice at the margin.

#### 4.10.2. Alternative sample restrictions.

Additional robustness checks restrict the sample to post-2000 waves and to high-coverage countries with longer panels. Table 16 shows that persistence, integration, and gender egalitarian coefficients remain positive and broadly similar across samples, with moderate variations in magnitude.

**Table 16:** Robustness checks: alternative sample restrictions. *Dependent variable: National wellbeing climate.*

Variables	Full sample	Post-2000 sample	High-coverage countries
Lagged wellbeing climate	0.52*** (0.08)	0.49*** (0.09)	0.54*** (0.08)
Social integration climate	0.17* (0.07)	0.19* (0.08)	0.16* (0.07)
Gender egalitarianism	0.11* (0.05)	0.13* (0.06)	0.10* (0.05)
Controls	Yes	Yes	Yes
Survey-era FE	Yes	Yes	Yes
Observations	238	164	202
Countries	52	41	34
Instruments	31	28	29

Note. Entries are coefficients with standard errors in parentheses. All models include controls and survey-era fixed effects. The post-2000 and high-coverage columns apply the alternative sample restrictions described in Section 4.10.2. † p < .10, * p < .05, ** p < .01, *** p < .001.

The reported sample restrictions yield coefficients with similar signs, but the modest magnitudes and overlapping uncertainty intervals limit claims of stability. These checks do not address the changing-domain composite or the three-wave integration constraint.

### 4.11. Dynamic panel diagnostic tests and instrument validity

Table 17 reports AR(1), AR(2), Hansen, and Difference-in-Hansen tests for representative specifications.

**Table 17:** System GMM diagnostic tests.

Model	AR(1) p-value	AR(2) p-value	Hansen J p-value	Difference-in-Hansen p-value	Instruments	Countries
Baseline dynamic model	0.02	0.29	0.34	0.41	28	52
Moderation model	0.03	0.26	0.31	0.38	31	49
Gender domain model	0.02	0.27	0.36	0.43	31	52
Environmental domain	0.03	0.32	0.39	0.46	27	46

Note. Entries are p-values. Significant AR(1) and nonsignificant AR(2) results are consistent with the reported instrument strategy. Hansen and Difference-in-Hansen tests assess the stated moment conditions but do not establish measurement invariance, contemporaneous exogeneity, or absence of residual confounding.

The reported AR(1) tests are significant and AR(2) tests are not. Hansen and Difference-in-Hansen tests do not reject the stated instrument sets, and instrument counts are below the number of countries. These diagnostics assess selected moment conditions; they do not demonstrate contemporaneous exogeneity of integration, measurement invariance, or absence of residual confounding. Table 18 summarizes the empirical status of the main hypotheses.

**Table 18:** Empirical status of the main hypotheses.

Test	Prediction	Result
H1: Wellbeing persistence	Positive lagged effect	Supported
RQ1: Domain-specific modernization associations	No directional prediction	Heterogeneous and modest across domains
H3: Social integration is positively associated with wellbeing	Positive association	Supported; modest magnitude
H4: Integration moderates modernization effect	Positive interaction	Consistent with prediction; moderate magnitude

Note. Classifications summarize the reported conditional associations “Supported” does not imply a causal effect. RQ1 is nondirectional and is evaluated through differences across modernization domains.

### 4.12. Hypothesis evaluation summary

The reported results support H1 as persistence in relative wellbeing positions, address RQ1 with heterogeneous domain estimates, support the directional association in H3 with a modest coefficient, and are consistent with the positive interaction in H4. Each conclusion is qualified by measurement, temporal-coverage, and endogeneity limits.

### 4.13. Empirical synthesis and interpretation

Taken together, the reported estimates show persistence in relative national wellbeing positions. Integration is positively associated with wellbeing, with coefficients near one-sixth of a standard deviation. Modernization associations differ across domains, and the positive interaction is moderate. These patterns are conditional macro associations and cannot distinguish contextual effects from respondent composition or establish causal direction.

## 5. Discussion

### 5.1. Normative climates, integration, and the psychological structure of collective wellbeing

The reported analysis yields three bounded conclusions. Relative national wellbeing positions persist across module periods. Social integration is positively but modestly associated with wellbeing. Modernization estimates differ by domain and become more positive at higher integration values. Low between-country variance, sparse integration measurement, changing composite content, and observational identification prevent stronger contextual or causal claims.

The strong persistence observed in wellbeing climates is consistent with theoretical perspectives emphasizing that collective psychological environments reflect durable institutional and normative conditions rather than purely contemporaneous contextual factors [16,33,57]. From a social psychological perspective, persistence reflects continuity in shared expectations, legitimacy perceptions, and meaning structures that shape how individuals interpret and experience social environments. Normative climates evolve through cumulative processes of institutional reinforcement and social coordination, generating stability in collective psychological conditions over time [8,45]. This persistence highlights that collective wellbeing is embedded in broader normative and structural contexts that cannot be understood solely through contemporaneous social change.

Social integration is the most consistent reported correlate, but the coefficient of about 0.16 to 0.18 is modest relative to the lagged wellbeing coefficient near 0.50. The association is compatible with evidence linking social connection to support and predictability, yet bidirectionality remains plausible: contemporaneous wellbeing shocks may also alter participation and relationships. The estimates should therefore be read as conditional correlations rather than as the effect of increasing integration.

Normative modernization indicators have heterogeneous and generally modest reported associations. The null inequality-attitude estimate may reflect a gap between preferences for fairness and experienced material conditions. The null work-modernization estimate may reflect differences in labor-market security, informality, job quality, or development stage that are not measured by the aggregate attitude scale. These explanations are plausible boundary conditions, not tested mechanisms. The changing item composition and the work scale's alpha of 0.68 also make a substantive null difficult to distinguish from measurement attenuation.

The domain pattern may also differ across institutional settings. In lower-security labor markets, autonomy-oriented work values may coexist with unemployment risk or weak social protection, while similar values in higher-security markets may carry different wellbeing implications. Inequality attitudes can likewise reflect either legitimation of existing differences or dissatisfaction with distributive institutions. Testing these alternatives requires country-level labor-market and development measures and interactions that are not included here.

The positive interaction is consistent with the proposed conditional framework, but it is not direct evidence of the mechanism. Integration may stabilize expectations, yet the macro design does not observe perceived consensus, credibility, or institutional embedding. The result identifies a conditional association whose mechanism remains inferential.

### 5.2. Theoretical implications for social psychology of norms and collective environments

The findings qualify social psychological accounts of norms by showing that aggregate normative indicators do not have uniform associations with aggregate wellbeing. The country-wave design, however, cannot demonstrate that national means influence individual outcomes beyond individual characteristics. Accordingly, ‘climate’ denotes a theoretical interpretation of aggregate indicators, not an independently identified contextual effect.

The reported conditional pattern is compatible with theories in which social integration stabilizes expectations, but the analysis does not observe that mechanism. It cannot determine whether the interaction reflects contextual influence, respondent composition, measurement differences, or another country-level process. The result therefore narrows the empirical claim to a positive aggregate interaction in the reported specification.

The domain results extend the descriptive comparison beyond individual attitudes by examining country-wave means. They do not demonstrate that a shared national normative environment shapes individual wellbeing. A multilevel analysis with individual covariates and country-level predictors would be required to separate that contextual interpretation from composition.

The reported integration coefficient is consistent with theories linking social connection to support and predictable interaction. The macro association does not establish that an integration climate produces psychological stability, because contemporaneous reverse association and compositional differences remain plausible.

The domain-specific pattern helps narrow competing claims about modernization. Some reported associations are positive, while others are null or imprecise. The evidence does not show that modernization is necessary or sufficient for wellbeing; it indicates that aggregate associations depend on domain, measurement, and integration level.

### 5.3. Dynamic normative climates and the temporal structure of collective psychological environments

The reported lag coefficient indicates persistence in countries’ relative wellbeing positions across module periods. It is compatible with institutional and normative continuity, but within-wave standardization and the aggregate design prevent interpreting it as continuity in absolute wellbeing or proof of a shared psychological environment.

The reported dynamic specification treats normative indicators as slow-moving predictors, yet it does not observe the timing of individual psychological responses. The positive interaction may reflect gradual institutional embedding, respondent composition, or other country-level processes. The present design cannot adjudicate among these explanations.

Including a lagged standardized outcome separates some persistence in relative country positions from contemporaneous association. It does not recover an absolute wellbeing trajectory, resolve all reciprocal relationships, or eliminate time-varying confounding.

### 5.4. Methodological and conceptual contributions of the climate framework

The aggregation strategy provides a macro description of country-wave means, but it cannot separate contextual influence from composition. A multilevel design linking country indicators to individual wellbeing while controlling individual covariates would be required to test a contextual climate effect directly.

Treating modernization as multidimensional prevents the changing-domain composite from carrying the full inference. The domain-specific estimates show different directions and levels of precision. Because the composite averages different domain sets across observations, it remains exploratory rather than a fixed measure of one longitudinal construct.

The climate framework is retained as a theoretical interpretation of the aggregate pattern. The reported interaction is consistent with, but does not identify, an expectation-stabilization process. Competing explanations based on respondent composition, institutional conditions, or measurement differences cannot be separated in the present design.

The policy implication is limited but concrete. Governments should not assume that attitudinal modernization alone will improve population wellbeing. Reforms concerning gender equality, environmental protection, work, or redistribution can be accompanied by investments in accessible civic organizations, community services, inclusive consultation, labor-market security, and reliable public institutions. Monitoring should track both wellbeing and social connection during periods of reform. The analysis does not identify individual countries as causal cases, so country-specific prescriptions would exceed the evidence.

### 5.5. Limitations and directions for future research

Several limitations materially restrict inference. First, ICC(1) values of 0.07 to 0.13 show that most respondent variation occurs within rather than between countries. Sizeable ICC(2) values reflect precision of country means but do not establish homogeneous national climates. The analysis also lacks individual-level multilevel tests, so aggregate associations may be compositional.

Second, full cross-national measurement invariance was not established. Translation, response styles, item thresholds, and cultural interpretation may affect comparisons, especially between culturally distant countries. Pooled reliability and CFA results cannot rule out these sources of differential measurement.

Third, the composite modernization indicator changes with the domains observed in each country-wave. It is not a fixed longitudinal scale. Composite estimates are therefore exploratory, and domain-specific estimates define the defensible scope of the modernization conclusion.

Fourth, integration is directly observed in only 1986, 2001, and 2017. Few countries contribute all three observations, limiting dynamic identification and making the interaction sensitive to harmonization. Treating integration as predetermined may also be incorrect if wellbeing shocks affect social relationships contemporaneously; a stable fully endogenous sensitivity model could not be estimated from the retained prepared data.

Fifth, within-module-wave standardization expresses relative country positions rather than absolute change. The lag coefficient therefore measures rank persistence across different cross-national distributions. Broad survey-era indicators cannot absorb shocks within each multi-year period.

Sixth, participating countries are concentrated in higher-income Western and Eastern Europe, with uneven representation elsewhere. The results should not be generalized to low-income countries or regions with different institutional, religious, labor-market, or family systems without additional evidence.

Finally, the observational macro panel cannot eliminate reverse association, omitted variables, selection into ISSP participation, or residual measurement error. The reported estimates describe conditional associations and do not identify causal effects or the proposed psychological mechanisms.

## 6. Conclusion

The reported evidence supports a narrower claim than the original climate framework proposed. Countries’ relative wellbeing positions are persistent, integration is positively but modestly associated with wellbeing, and modernization associations vary across domains and integration levels. The design does not establish causal effects or contextual influence beyond respondent composition.

The theoretical implication is that aggregate normative change should not be treated as a uniform predictor of wellbeing. Measurement comparability, domain content, social connection, and institutional setting define the boundary of the observed associations.

What remains unresolved is whether changes in social integration and normative environments precede changes in individual wellbeing, or instead reflect the same institutional and compositional processes.

Answering that question requires comparable repeated measures, deeper integration panels, and designs that connect national indicators to individual outcomes.
